# Critical limb ischaemia in a diabetic population from an Asian Centre: angiographic pattern of disease and 3-year limb salvage rate with percutaneous angioplasty as first line of treatment

**DOI:** 10.2349/biij.6.4.e33

**Published:** 2010-10-01

**Authors:** M Tan, U Pua, DES Wong, SJ Punamiya, GC Chua, N Teo

**Affiliations:** 1 Diagnostic Radiology, Tan Tock Seng Hospital, Singapore; 2 Diagnostic Radiology, Gleneagles Hospital, Singapore; 3 Diagnostic Radiology, Mt. Alvernia Hospital, Singapore

**Keywords:** Limb salvage rate, lower limb vascular disease pattern, diabetics, Asian centre, percutaneous angioplasty

## Abstract

**Purpose:**

Lower extremity amputation prevention (LEAP) is an ongoing program in our institution aimed at salvaging limbs in patients with critical limb ischemia (CLI). Patients in the LEAP program with reconstructible anatomy on initial Doppler imaging received either bypass surgery or percutaneous transluminal balloon angioplasty (PTA). We present the 3 year limb salvage rate and angiographic disease patterns in 42 consecutive diabetic patients with CLI who received PTA in 2005.

**Methods and Material:**

26 women and 16 men with diabetes between the ages of 45 and 91 years old (mean age, 70.8 years) received PTA in 2005. Presenting symptoms were rest pain (n = 22), pre-existing gangrene (n = 17), non-healing ulcer (n = 16) and cellulitis (n = 2). The aim of the PTA was to achieve straight-line flow from the abdominal aorta down to the patent dorsalis pedis or plantar arch, with limb salvage as the ultimate outcome. Failure of treatment was defined as any amputation above the level of a Syme’s amputation or the need for further surgical bypass. Technical success was achieved in 90% (38 out of 42 patients).

**Results:**

Limb salvage rates were 93% at 1 month, 87% at 3 months, 82% at 6 months, 78% at 1 year, 69% at 2 years and 66% at 3 years. Mortality was 17% (n = 7) at 3 years. Of the 13 patients with failed therapy, 3 underwent bypass, 9 had amputations and 1 had bypass followed by amputation. Four of the cases required further intervention due to worsening gangrene and infection, while the remaining was due to persistent rest pain. The rest of the 32 patients had no lower limb related issues at the end of 3 years, with improvement of the presenting symptoms. Patterns of treated segments were aortoiliac occlusions (n = 3), pure infrapopliteal disease (n = 3), femoropopliteal with at least 1 good infrapopliteal run-off vessel (n = 14) and combined femoropopliteal and infrapopliteal disease (n = 25).

**Conclusion:**

Involvement of infrapopliteal vessels that needs to be treated is common in Asian diabetics. While early limb salvage rates up to 1 year are similar, the 3 year limb salvage rates in Asian diabetics are lower than the western population.

## INTRODUCTION

Diabetes mellitus (DM) is a multisystem disorder often associated with peripheral vascular disease. The disease is a common cause of small vessel vasculopathy, hence accounting for the myriad forms of clinical presentation. The described pattern of atherosclerotic disease is often more diffuse in diabetics, with more severe involvement of the distal small vessels.

Critical limb ischemia (CLI) is the most severe form of peripheral vascular disease where there is inadequate blood flow to a limb to maintain reasonable metabolic requirement of tissues at rest. The lower extremity amputation prevention (LEAP) is a comprehensive program aimed at reducing lower extremity amputations in individuals with peripheral vascular disease. The aims of LEAP are to detect and treat early chronic ischaemic lesions, offer alternatives to amputations in surgically untreatable patients, preserve the remaining limb after major amputation and reduce the global risk for cardiovascular disease. Hence, it is a multidisciplinary program with involvement at various levels from risk factor modification and patient education at the primary healthcare level, to active treatment of critical limb ischaemia at tertiary institutions.

Critical limb ischaemia may be treated conservatively. However, if conservative therapy does not lead to improvement (showing symptoms like increasing wound size, and persistent or spreading infection), intervention in the form of percutaneous transluminal angioplasty (PTA) or bypass surgery should be done. It has been described that critical limb ischaemia has a three-year limb loss rate of 40% [1] with conservative therapy.

There have been few studies describing the pattern of lower limb peripheral vascular disease in a diabetic population, especially the Asian diabetic population. We aim to describe the pattern of the vascular disease in an Asian Diabetic population, as well as 3 years limb salvage rates in the these patients, treated by PTA, in the LEAP program.

## MATERIALS AND METHODS

### Patient Selection

Since the inception of LEAP in 2001 up to 2005, a total of 301 patients with CLI have been enrolled in this study. Patients presenting with CLI to the authors’ institution first received Doppler vascular assessment of both lower limbs to assess the anatomical reconstructibility of the lower limb arteries. Those with reconstructible anatomy received either bypass surgery or percutaneous transluminal angioplasty (PTA). The patients with non-reconstructible anatomy received hyperbaric oxygen, lumbar sympathectomies or pneumatic compression. Patients who were unfit for any of the above received primary amputation and were put under close surveillance for the remaining limb.

### Percutaneous Transluminal Angioplasty (PTA) Arm

In 2005, a total of 46 consecutive patients with CLI were enrolled into the PTA treatment arm. The period of the study was from 1 January 2005 to 1 January 2008, a period of three years. All the patients were recruited and followed-up in the study period. The time of entry into the study was taken as the date of the first angioplasty.

The patients were enrolled in the PTA treatment arm based on the following inclusion criteria: 1) patients who were medically unfit for bypass surgery; 2) patients who were fit for bypass surgery but chose to have PTA; 3) All patients must either have patent dorsalis pedis or a patent planter arch on the baseline Doppler vascular maps. Of the enrolled 46 patients, 42 were diabetic.

**Figure 1:**
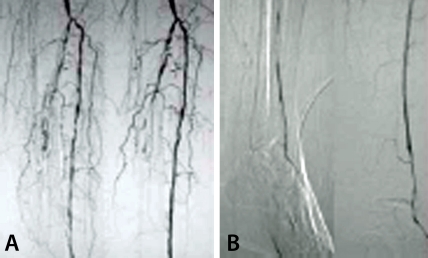
Pre and Post Angioplasty runs in a 70-Year-Old Diabetic Male

The aim of the PTA was to achieve straight-line flow from the abdominal aorta down to the patent dorsalis pedis or plantar arch, with limb salvage as the ultimate outcome. Technical success was defined as achieving straight-line flow from the aorta into any intrapopliteal vessel that supplies either a patent dorsalis pedis or plantar arch via collateral reconstitution (Figure 1).

Extensive atherosclerotic disease with irregular lumen in opacified vessels seen in pre and post –percutaneous transluminal angioplasty (PTA) angiograms. Both Figure A and B are below knee angiograms, with the image on the left, being the pre-angioplasty run and those on the right the post angioplasty run. In the pre-PTA angiogram, there is poor infrapopliteal single vessel (anterior tibial artery) run-off into the foot. Long occlusions of the peroneal and posterior tibial arteries are also present bu not angioplastied. The post-PTA angiogram demonstrated technical success in achieving “straight-line flow” into the dorsalis pedis.

Technical failure was defined as any subsequent amputations above the level of a Syme amputation or the need for surgical bypass. All the patients who were enrolled into the PTA treatment arm received PTA as the only form of treatment (apart from medical therapy for risk factor modification).

All the angioplasties were performed by one of three interventional radiologists in the department. Antegrade arteriotomy was preferred and performed in all patients who had no evidence of aorto-iliac disease on Doppler arterial map. Vascular access was secured with a 5.5 F vascular sheath (Terumo Corporation, Tokyo, Japan) in all cases. Intra-arterial heparin 3000 IU was routinely administered via the vascular sheath at the start of the procedure and intra-arterial glycerin Trinitrate 200 mcg was administered before all tibial angioplasties. 0.035″ wires (Terumu Corporation, Tokyo, Japan) were used to cross most femoropopliteal lesions while smaller diameter 0.018″ wires (V-18) control wire (Boston Scientific) was used to cross infrapopliteal lesions. Balloons used for angioplasties included: 5 × 40 mm and 5 × 100 mm 5F (Ultrathin, Boston Scientific) for most superficial femoral artery lesions; and 3 × 40 mm and 3 × 100 mm 3F microballoons (Savvy, Cordis/Johnson & Johnson) for most infrapopliteal lesions. The decision about which balloon to use was based on the length of the lesion. Short lesions were treated with the shorter balloon, while the longer lesions were treated with the longer balloon. Only one type of balloon was used for any particular lesion. Dissections, if any, received first-line treatment with prolonged balloon inflations. All vascular sheaths were removed two hours after angioplasty and homeostasis was controlled by manual compression for at least ten minutes. Antiplatelet therapy was started immediately post-PTA using aspirin 100 mg or clopidogrel bisulfate 75 mg for patients in whom aspirin was contraindicated.

### Follow-up

ABI (Ankle Brachial Index), TP (Toe Pressure) and digital-brachial index (DBI) measurements were also obtained before angioplasty and on the first day post-angioplasty. Post-procedure surveillance was conducted in the form of three-monthly clinic reviews by the referring vascular surgeon. ABI, as well as relevant indicators of clinical improvement like wound healing and rest pain, was evaluated. The patients were kept under surveillance for periods ranging from 25 to 35 months, with a mean of 31.2 months.

### Outcome Assessment

The study describes the pattern of the vascular disease as well as the three-year limb salvage rates in the subgroup of diabetic patients, treated by PTA, in the LEAP program. All the patients were recruited in 2005, and followed up to 2008, a total of three years. The one-month, three-month, six-month, one-year, two-year and three-year limb salvage rates were studied.

### Statistical Analysis

SPSS software was used for statistical analysis. Paired T tests were performed to determine if there was any statistical difference in the improvement of the measures of haemodynamic markers within the technically successful group. Kaplan-Meir analysis of both the limb salvage rate, as well as a combined salvage and survival rate was performed.

## RESULTS

**Figure 2:**
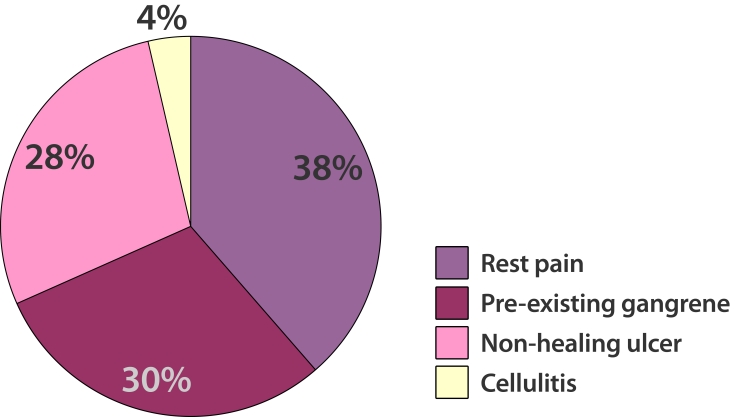
Range of presenting symptoms

There were 42 patients studied, of which 26 were women and 16 were men, between the ages of 45 and 91 years (mean age, 70.8). The ethnic distribution was 76% Chinese, 10% Malays and 14% Indians. Common presenting symptoms were rest pain (n = 22), pre-existing gangrene (n = 17), non-healing ulcer (n = 16) and cellulitis (n = 2) (Figure 2).

**Figure 3:**
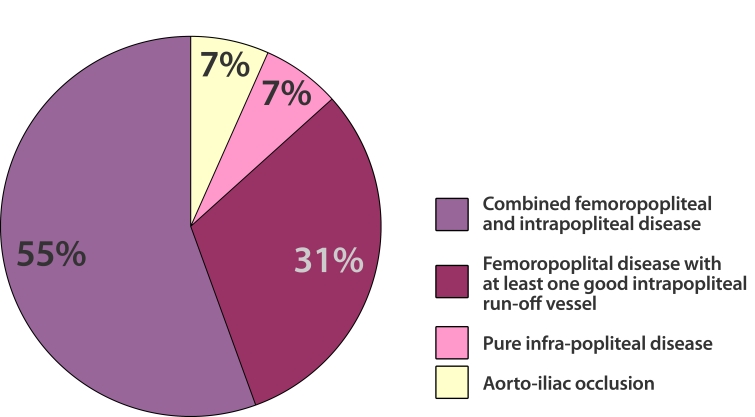
Patterns of Treated diseases

A total of 40 femoral, one brachial and one popliteal arteriotomy were performed and the angiograms obtained. Patterns of treated segments were aorto-iliac occlusions (n = 3), pure infra-popliteal disease (n = 3), femoropopliteal with at least 1 good intrapopliteal run-off vessel (n = 14), and combined femoropopliteal and intrapopliteal disease (n = 25) (Figure 3). A summary of the locations of the chronic total occlusions and severe stenosis is shown in Table 1.

**Table 1:** Summary of locations of the chronic total occlusions and severe stenosis

	Superficial femoral artery	Infrapopliteal
Chronic total occlusion	N = 15, range 1 to 25 cm, mean 8.13 cm	N = 21, 1 to 23 cm, mean 10.1 cm
Severe stenosis (> 70% stenosis)	N = 26, range 1 to 30 cm, mean 9.34 cm	N = 23, 2 to 25 cm, mean 8.73 cm

Technical success was achieved in 90% of the patients (38 out of 42 patients). The most common cause of technical failure was inability to cross long chronic occlusions (CTOs). The locations of the CTOs in the failed cases were the anterior tibial artery (n = 3; 15, 20 and 23 cm in lengths) and complete distal posterior tibial (n = 1). In all 4 technical failures, subintimal PTA was attempted with failed re-entry.

Transluminal angioplasty was performed in 35 patients. Both transluminal and subintimal angioplasty were performed in 4 patients. Three patients received subintimal angioplasty. Only in one case was an endovascular stent deployed (over an iliac stenosis). In the technically successful group, repeat interval angioplasties were performed on 4 patients at 6 weeks (n = 2), four months (n = 1) and seven months (n = 1).

Paired T tests showed statistical improvement in the haemodynamic markers within the technically successful group. This included mean ABI improvement from 0.60 (pre-angioplasty) to 0.91 (day one post-angioplasty), which was a significant increase of 0.31 [95% confidence interval (95%); CI, 0.20 to 0.41; P < 0.001]. There was also a 0.18 increase in the mean DBI, from 0.21 to 0.38 (pre-angioplasty versus day one post-angioplasty), which was also noted to be significant [95% CI of 0.11 to 0.24; P < 0.001]. There was also a significant increase in TP from 30.2 to 59.6 (pre-angioplasty versus day one post-angioplasty), with a 95% CI of 20.2 to 38.2 (P < 0.001).

Within the technically successful group, repeat angioplasty was performed on four patients. The reasons for the repeat angioplasty included decrease in ABI by more than 0.15 (n = 2) and persistent non-healing of the wound (n = 2) during the follow-up period. Of these four patients, only one went on to have surgical bypass six months after the first angioplasty for persistent rest pain. This patient subsequently had an above-knee amputation, as the rest pain was still not resolved. Among the technical failures (n = 4), two went on to receive amputations (both below-knee amputations), one went on to have a successful bypass operation, and one died of an unrelated cause three months later (necrotising fasciitis), and had both the lower limbs intact at the time of death.

Within the technically successful group, two went on subsequently for successful bypass surgeries, nine went on for amputations; five of which were above-knee amputations, two were below-knee amputations and two were forefoot amputations.There were no instances of significant distal embolisation, arterial perforations or procedure-related deaths (30-day post-procedural mortality). All intimal dissections encountered were successfully treated with prolonged balloon inflations, with no case needing bail-out stenting.

PTA-related complications that were encountered included one case of early arterial thrombosis (requiring an above-knee amputation after failed thrombolysis), three cases of groin haematomas (resolved with conservative treatment) and one case of pseudoaneurysm (treated with ultrasound-guided compression).

**Figure 4:**
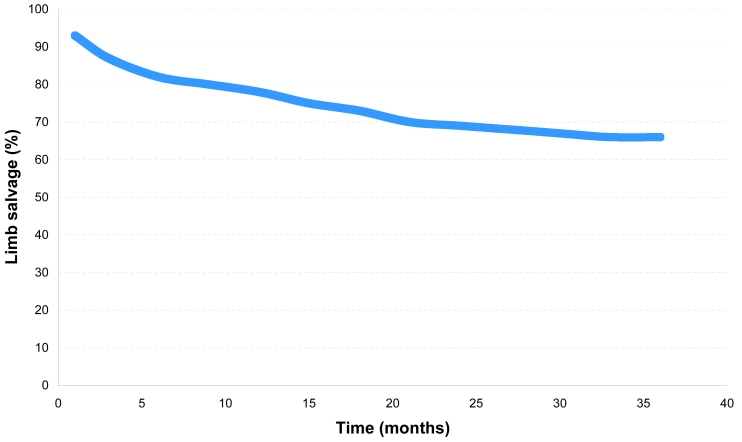
3 year limb salvage rate: Change in limb salvage rates against time (months)

**Figure 5:**
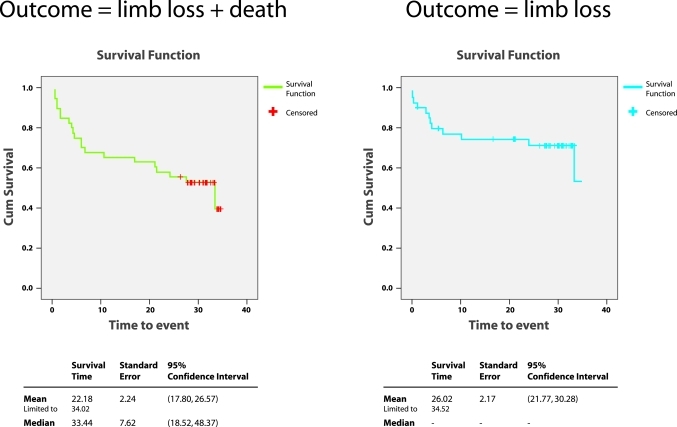
Kaplan-Meier curve showing the relationship of limb loss and death over time.

Limb salvage rates were 93% at one month, 87% at three months, 82% at six months, 78% at one year, 69% at two years and 66% at three years (Figure 4). Minor amputations up to the level of Syme amputation were not considered limb loss. Within the study period, there were six deaths from unrelated causes (sepsis, pneumonia and necrotising fasciitis of the elbow). More importantly, all six patients had intact limbs at the time of the death. Kaplan-Meir analysis of both the limb salvage rate, as well as a combined salvage and survival rate is illustrated (Figure 5). Clinically, of the 22 patients who presented with rest pain, total abolishment of the symptoms was achieved in 20 patients (91%). 17 patients presented with pre-existing gangrene, for which healing was seen in 11 patients (66%). Within the technically successful group, four patients went on to have minor amputation (ray amputation) for pre-existing gangrene. Clinical improvement in the patients who presented with non-healing ulcers (partial or complete healing) and cellulitis was obtained. More importantly, all healed ulcers remained healed throughout the study period.

## DISCUSSION

Almost all the diabetic patients seen in the authors’ centre have extensive infra and supra-popliteal disease. There has been another study described in the literature [2], which demonstrates that vasculopathy, namely stenosis, tortuosity, mural irregularity and calcification of the arterial walls, is more frequent in patients with diabetes than those without. However, these were often non-occlusive lesions, particularly calcification along the arterial walls. Most of the patients in that study also had disease involving both the femoral and infrapopliteal vessels. They found that patients with diabetes had arterial occlusions less frequently than non-diabetic patients. They concluded that more attention should be paid to morphologic lesions of arteriosclerosis other than occlusions or stenosis, and also concluded that more studies were needed to study this topic. Neubauer *et al.* [3] also concluded that large vessels of the muscular type, progressive and uniform narrowing, increased intima roughness or rugosity, and linear media calcification were related to type 1 diabetes. Another study by Graziani *et al.* [4] also showed that vascular disease in diabetic patients tends to involve the distal calf vessels.

Hence, lower limb peripheral vascular disease is more common in diabetics, with extensive involvement of the entire lower limb. However, whether they are clinically significant is subject to further research.

The ethnic distribution of this study was 76% Chinese, 10% Malays and 14% Indians. The ethnic distribution in Singapore is 77% Chinese, 14% Malays and 7% Indians. Hence there was a disproportionately higher percentage of Indians in the study. It is also important to note that the Indians in this study were predominantly Asian Indians. Studies have demonstrated that one important factor contributing to increased type 2 diabetes among Asian Indians is excessive insulin resistance compared to Caucasians [5]. These studies have commented that the difference in the degree of insulin resistance may be explained by either environmental or genetic factors or a combination of both. Another study has postulated that Asian Indians often have increased visceral fat related to dyslipidaemia, and hence, increased frequency of insulin resistance, and this may account for the prevalence of diabetes mellitus and cardiovascular disease in Asian Indians [6].

The reported success rate for endovascular treatment of CLI is around 92% to 96%, with a one-year limb salvage rate of 68.6% to 90% [7–11]. This study, which is only confined to diabetic patients, shows a one-year limb salvage rate of 78%, which is comparable to those other studies. The published three-year limb salvage rate of PTA is around 77% to 94% [12–14]. The three-year limb salvage rate of this study is 66%, which makes it lower than the other studies. This could be attributed to the fact that this study involved a diabetic population, whereas most of the other studies involved a general population. It has been estimated that 40% to 45% of all amputees are diabetic [15], and a diabetic patient with CLI is more likely to require an amputation compared to a non-diabetic [16].

DM affects CLI in many ways. Premature and advanced atherosclerosis, together with peripheral neuropathy, impaired cellular immunity and impaired wound healing make CLI a complex problem among diabetic patients. An aggressive approach must be taken to achieve optical glycaemic control. Hence, in this LEAP program, all the patients received review and assessment by an endocrinologist. Even under these circumstances, preferential involvement of the distal calf vessels, which tends to be more severe, can cause technical problems during PTA, explaining the relatively lower three-year limb salvage rate compared to other studies.

Surgical bypass, as well as endovascular re-vascularisation, are currently accepted as methods of treatment for CLI [13, 17]. Surgical re-vascularisation, in the form of arterial bypass, has traditionally been the main treatment for CLI with a well-documented long-term patency and limb salvage rate [18]. In most surgical series, the three-year bypass rates of calf arteries ranged from 40% for prosthetic bypasses to 85% for saphenous bypasses [7–9, 19, 20]. However, there are limiting factors, such as availability of long vein-graft and the presence of infection near the site of planned distal anastomosis, that make surgery challenging. Moreover, the added anaesthetic risk to patients in surgical bypass makes PTA an attractive alternative. A recent study by Fowkes *et al.* [21] described that the mortality and amputation rates did not differ significantly between bypass and PTA, though primary patency was significantly higher in the bypass group after 12 months, but not after four years. Moreover, in the patients with lower CLI, surgery was associated with increased surgical complications. The study concluded that there was limited evidence to recommend bypass surgery compared with other treatments, and that further large trials were needed.

During this study period, there were six deaths, but a significant finding is that these deaths were not related to CLI. Many of the patients had multiple co-morbidities, in addition to diabetes. They all died from sepsis secondary to other causes, and not from lower limb infection, which may be due to the immunocompromised state of the diabetics. It is known that in diabetic patients, co-existing cardiovascular morbidity and mortality, as well as renal impairment, pose an even greater risk to mortality than CLI.

The life expectancy of diabetics is often limited. Hence, although the long-term patency of angioplasty is not as good compared to arterial bypass surgery, if limb salvage is the ultimate goal, PTA would be an attractive option for these patients. Notably, the clinical results and limb salvage after PTA are known to be higher than the haemodynamic patency rate and it has been repeatedly shown that healed ischaemic lesions do not recur even with restenosis of the dilated vessels. In this study period, all the patients who presented with non-healing ulcers showed documented healing of the ulcers after PTA. Therefore, less blood flow is needed to keep tissues healed than to achieve healing. Surgical bypass patency, on the other hand, always exceeds the limb salvage rate [22]. In addition, most of the patients also showed improvement in the presenting symptoms of pain or gangrene.

Hence, in diabetic patients where life expectancy is limited and the aim is to preserve limb function, angioplasty would be a very attractive option. There have been several studies which demonstrate the attractiveness of PTA. A retrospective study by Kudo *et al.* [13] showed that angioplasty can replace open surgery without compromising the outcome. They concluded that angioplasty is not only safe and feasible, but is also an effective procedure and is the procedure of choice for the primary and secondary treatment of CLI. The Bypass versus Angioplasty in Severe Ischaemia of the Leg (BASIL) multicentre trial [19] also showed further that a bypass-surgery-first and a balloon-angioplasty-first strategy in suitable candidates with infra-inguinal disease are associated with broadly similar outcomes in terms of amputation-free survival, and in the short term. Furthermore, the perimortality rate of PTA compares favourably at around 1% – 3% [19, 20] to 1.8% – 6% for distal bypass surgery [7, 20].

### Limitations

Limitations in this study include potential confounders such as adherence to diabetic regimes and risk factor modification which have been shown to be equally important in altering the outcome in CLI. The lack of anatomical evidence (e.g. Doppler) for long-term vascular patency is also one limitation. However, if the patient did not show any recurrence of initial symptoms, it was taken that there was probably no need for anatomical evidence. Lastly, the follow-up time frame of three years may be relatively short, and further follow-up may be necessary to evaluate the long-term outcome. However, this study still demonstrated that PTA is useful for patients over the three-year outcome.

## CONCLUSION

Peripheral vascular disease in diabetics is severe and extensive. The most common pattern involves both the femoral and infrapopliteal vessels, with a high incidence of infrapopliteal disease that needs to be treated. First-year limb salvage rates are similar to those of other studies, but the three-year limb salvage rate is less, probably due to more extensive peripheral vascular disease. Diabetics with CLI have a limited life expectancy and often die from a non-limb-related cause. Hence, PTA is a good and viable option for these patients, where additional anaesthetic and surgical risks may not make bypass the best option. The three-year limb salvage rate in this study is 66%, which makes PTA an attractive option for the treatment of CLI in diabetic patients.
